# 1-(2-Aza­niumyleth­yl)piperazine-1,4-diium trinitrate

**DOI:** 10.1107/S1600536811047507

**Published:** 2011-11-12

**Authors:** M. Rademeyer

**Affiliations:** a Department of Chemistry, University of Pretoria, Pretoria, 0002, South Africa

## Abstract

In the title salt, C_6_H_18_N_3_^3+^·3NO_3_^−^, the piperazine ring adopts a chair conformation and the ethyl­ammonium group is equatorial relative to the piperazine ring, and in an all-*trans* conformation. In the crystal, strong charge-assisted N—H⋯O hydrogen bonds link the piperazinediium trications and the nitrate anions into a three-dimensional network

## Related literature

The structure of a related salt, bis­(1-(2-ammonium­eth­yl)piperazinium) cyclo­hexa­phosphate hexa­hydrate, has been reported (Charfi & Jouini, 1996 ▶).
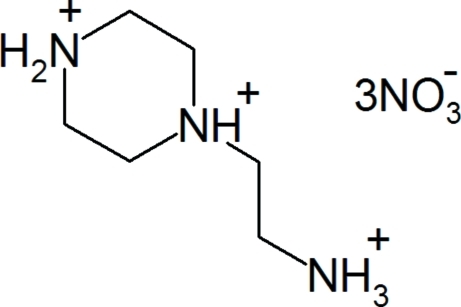


## Experimental

### 

#### Crystal data

C_6_H_18_N_3_^3+^·3NO_3_^−^
*M* *_r_* = 318.26
Monoclinic, 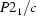

*a* = 7.7946 (6) Å
*b* = 8.9320 (6) Å
*c* = 19.6910 (13) Å
β = 96.635 (6)°
*V* = 1361.73 (17) Å^3^
*Z* = 4
Mo *K*α radiation
μ = 0.14 mm^−1^
*T* = 293 K
0.35 × 0.30 × 0.20 mm

#### Data collection

Oxford Xcalibur2 diffractometer
Absorption correction: multi-scan (*CrysAlis RED*; Oxford Diffraction, 2006 ▶) *T*_min_ = 0.966, *T*_max_ = 1.043
13603 measured reflections
4319 independent reflections
2517 reflections with *I* > 2σ(*I*)
*R*_int_ = 0.022

#### Refinement

*R*[*F* ^2^ > 2σ(*F* ^2^)] = 0.042
*wR*(*F* ^2^) = 0.129
*S* = 1.01
4319 reflections
192 parameters
H-atom parameters constrained
Δρ_max_ = 0.24 e Å^−3^
Δρ_min_ = −0.21 e Å^−3^

### 

Data collection: *CrysAlis CCD* (Oxford Diffraction, 2006 ▶); cell refinement: *CrysAlis RED* (Oxford Diffraction, 2006 ▶); data reduction: *CrysAlis RED*; program(s) used to solve structure: *SHELXS97* (Sheldrick, 2008 ▶); program(s) used to refine structure: *SHELXL97* (Sheldrick, 2008 ▶); molecular graphics: *Mercury* (Macrae *et al.*, 2008 ▶); software used to prepare material for publication: *PLATON* (Spek, 2009 ▶) and *WinGX* (Farrugia, 1999 ▶).

## Supplementary Material

Crystal structure: contains datablock(s) I, global. DOI: 10.1107/S1600536811047507/bt5713sup1.cif

Structure factors: contains datablock(s) I. DOI: 10.1107/S1600536811047507/bt5713Isup2.hkl

Supplementary material file. DOI: 10.1107/S1600536811047507/bt5713Isup3.cml

Additional supplementary materials:  crystallographic information; 3D view; checkCIF report

## Figures and Tables

**Table 1:** Hydrogen-bond geometry (Å, °)

*D*—H⋯*A*	*D*—H	H⋯*A*	*D*⋯*A*	*D*—H⋯*A*
N5—H5⋯O1^i^	0.91	2.00	2.8074 (16)	146
N5—H5⋯O2^i^	0.91	2.34	3.1755 (17)	152
N4—H4*A*⋯O9	0.90	2.05	2.9008 (15)	158
N4—H4*A*⋯O7	0.90	2.29	2.9947 (15)	135
N4—H4*B*⋯O5^ii^	0.90	1.93	2.8065 (16)	165
N4—H4*B*⋯O6^ii^	0.90	2.43	3.0387 (15)	125
N6—H7*A*⋯O1^iii^	0.89	2.16	2.9224 (15)	144
N6—H7*A*⋯O3^iii^	0.89	2.47	3.322 (2)	161
N6—H7*B*⋯O9^iv^	0.89	2.15	3.0360 (15)	173
N6—H7*B*⋯O8^iv^	0.89	2.45	3.1171 (16)	132
N6—H7*C*⋯O4^v^	0.89	1.99	2.8033 (14)	152

Symmetry codes: (i) 
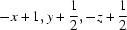
; (ii) 
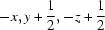
; (iii) 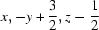; (iv) 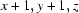; (v) 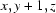.

**Fig. 1.**
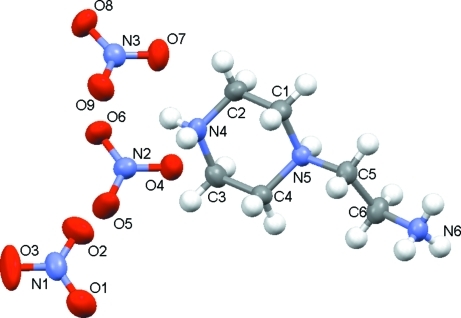
The asymmetric unit of I, showing the atomic numbering scheme. Displacement ellipsoids are shown at the 50% probability level and H atoms are shown as small spheres of arbitrary radii.

**Fig. 2.**
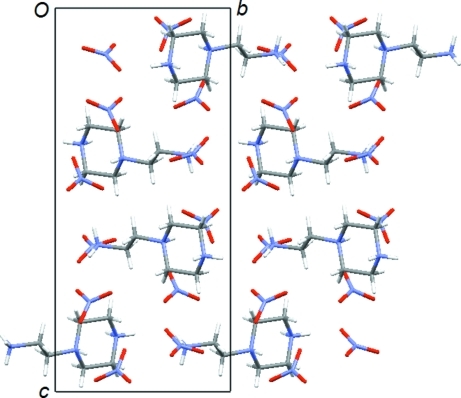
Packing diagram of I viewed down the a-axis.

**Fig. 3.**
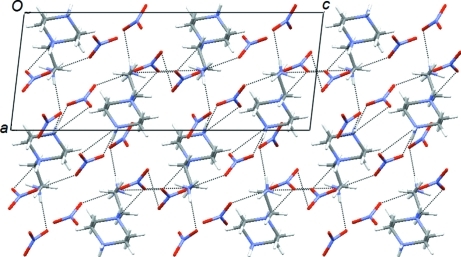
Hydrogen bonding network in I.

## supplementary crystallographic information

### Comment

In the field of crystal engineering, an understanding of the role of the anion geometry on the molecular packing and non-covalent interactions in salt crystal structures is central to the area of molecular recognition. The current structure was determined as part of a wider study that considers this role of anion geometry on a crystal structure.

The molecular geometry and labelling scheme of 1-(2-ammoniumethyl)piperazinium) trinitrate, I, is illustrated in Fig. 1. In this structure, the asymmetric unit consists of one 1-(2-ammoniumethyl)piperazinium cation and three isolated, trigonal planar nitrate anions, with four asymmetric units in the unit cell. In the cation the piperazine ring adopts the chair conformation, and the ethylammonium group is equatorial relative to the piperazine ring, and in the all-*trans* conformation.

Fig. 2 shows the molecular packing of I, viewed down the *a*-axis, with double rows of cations alternating in orientation along the *c*-axis. Each cation is hydrogen bonded to six different surrounding nitrate anions, and strong N—H^+^···^-^O—N hydrogen bonds link the cations and the anions. The ammonium group on the cation froms one conventional and two bifurcated hydrogen bonds to three different nitrate anions, while the –NH_2_^+^ group is involved in two bifurcated hydrogen bonds to two nitrate anions, and the –NH^+^ group forms one bifurcated hydrogen bond to one nitrate anion. Hydrogen bonding interactions are listed in Table 1, and the resulting, complex, three-dimensional hydrogen bonding network is shown in Fig. 3.

### Experimental

1-(2-Ammoniumethyl)piperazinium trinitrate was prepared by the dropwise addition of excess concentrated nitric acid (3.0 ml, 0.047 mol, 70%, Saarchem) to a solution of 1-(2-aminoethyl)piperazine (1.5 ml, 0.011 mol 99%, Aldrich) in 40 ml chloroform (99%, Saarchem). The resulting precipitate was filtered, dried in air and re-crystallized from distilled water. Colourless crystals formed on evaporation, open to the air, at room temperature.

### Refinement

All H atoms were refined using a riding model, with C—H distances of 0.97 Å and N—H distances of 0.89 Å, and *U*_iso_(H) = 1.5*U*_eq_(C) or 1.2*U*_eq_(C) or 1.2*U*_eq_(N). The highest residual peak (0.24eÅ^-3^) is 0.83 Å from atom O1.

### Crystal data

**Table:**

C_6_H_18_N_3_^3+^·3NO_3_^−^	*F*(000) = 672
*M**_r_* = 318.26	*D*_x_ = 1.552 Mg m^−^^3^
Monoclinic, *P*2_1_/*c*	Mo *K*α radiation, λ = 0.71073 Å
Hall symbol: -P 2ybc	Cell parameters from 5277 reflections
*a* = 7.7946 (6) Å	θ = 3.7–32.0°
*b* = 8.9320 (6) Å	µ = 0.14 mm^−^^1^
*c* = 19.6910 (13) Å	*T* = 293 K
β = 96.635 (6)°	Block, colourless
*V* = 1361.73 (17) Å^3^	0.35 × 0.30 × 0.20 mm
*Z* = 4	

### Data collection

**Table:**

Oxford Xcalibur2 diffractometer	4319 independent reflections
Radiation source: fine-focus sealed tube	2517 reflections with *I* > 2σ(*I*)
graphite	*R*_int_ = 0.022
ω–2θ scans	θ_max_ = 32.0°, θ_min_ = 3.7°
Absorption correction: multi-scan (*CrysAlis RED*; Oxford Diffraction, 2006)	*h* = −11→6
*T*_min_ = 0.966, *T*_max_ = 1.043	*k* = −12→12
13603 measured reflections	*l* = −28→29

### Refinement

**Table:**

Refinement on *F*^2^	Secondary atom site location: difference Fourier map
Least-squares matrix: full	Hydrogen site location: inferred from neighbouring sites
*R*[*F*^2^ > 2σ(*F*^2^)] = 0.042	H-atom parameters constrained
*wR*(*F*^2^) = 0.129	*w* = 1/[σ^2^(*F*_o_^2^) + (0.074*P*)^2^] where *P* = (*F*_o_^2^ + 2*F*_c_^2^)/3
*S* = 1.01	(Δ/σ)_max_ = 0.001
4319 reflections	Δρ_max_ = 0.24 e Å^−^^3^
192 parameters	Δρ_min_ = −0.21 e Å^−^^3^
0 restraints	Extinction correction: *SHELXL97* (Sheldrick, 2008), Fc^*^=kFc[1+0.001xFc^2^λ^3^/sin(2θ)]^-1/4^
Primary atom site location: structure-invariant direct methods	Extinction coefficient: 0.033 (3)

### Special details

**Table:**

Experimental. CrysAlis RED (Oxford Diffraction, 2006) Version 1.171.29.9 Empirical absorption correction using spherical harmonics, implemented in SCALE3 ABSPACK scaling algorithm.
Geometry. All e.s.d.'s (except the e.s.d. in the dihedral angle between two l.s. planes) are estimated using the full covariance matrix. The cell e.s.d.'s are taken into account individually in the estimation of e.s.d.'s in distances, angles and torsion angles; correlations between e.s.d.'s in cell parameters are only used when they are defined by crystal symmetry. An approximate (isotropic) treatment of cell e.s.d.'s is used for estimating e.s.d.'s involving l.s. planes.
Refinement. Refinement of *F*^2^ against ALL reflections. The weighted *R*-factor *wR* and goodness of fit *S* are based on *F*^2^, conventional *R*-factors *R* are based on *F*, with *F* set to zero for negative *F*^2^. The threshold expression of *F*^2^ > σ(*F*^2^) is used only for calculating *R*-factors(gt) *etc*. and is not relevant to the choice of reflections for refinement. *R*-factors based on *F*^2^ are statistically about twice as large as those based on *F*, and *R*- factors based on ALL data will be even larger.

### Fractional atomic coordinates and isotropic or equivalent isotropic displacement parameters (Å^2^)

**Table:**

	*x*	*y*	*z*	*U*_iso_*/*U*_eq_	
O8	−0.12932 (14)	0.16141 (11)	0.10385 (6)	0.0633 (3)	
O9	−0.14426 (14)	0.36595 (11)	0.16044 (5)	0.0567 (3)	
N5	0.25593 (12)	0.88408 (10)	0.10913 (5)	0.0323 (2)	
H5	0.3465	0.8265	0.0997	0.039*	
N4	0.02701 (14)	0.65031 (12)	0.14525 (6)	0.0448 (3)	
H4A	0.0034	0.5530	0.1515	0.054*	
H4B	−0.0651	0.7043	0.1544	0.054*	
N6	0.49105 (14)	1.25521 (12)	0.11758 (5)	0.0432 (3)	
H7A	0.4563	1.2789	0.0743	0.065*	
H7B	0.6012	1.2814	0.1278	0.065*	
H7C	0.4265	1.3035	0.1449	0.065*	
C4	0.22499 (17)	0.85655 (14)	0.18187 (6)	0.0387 (3)	
H4C	0.1312	0.9198	0.1932	0.046*	
H4D	0.3278	0.8830	0.2120	0.046*	
C1	0.09846 (17)	0.83708 (15)	0.06264 (6)	0.0428 (3)	
H1A	0.0008	0.8981	0.0718	0.051*	
H1B	0.1182	0.8531	0.0154	0.051*	
C5	0.29617 (16)	1.04353 (13)	0.09492 (6)	0.0398 (3)	
H5A	0.2098	1.1071	0.1120	0.048*	
H5B	0.2890	1.0580	0.0458	0.048*	
C3	0.18042 (18)	0.69465 (15)	0.19308 (7)	0.0445 (3)	
H3A	0.2779	0.6317	0.1856	0.053*	
H3B	0.1561	0.6806	0.2399	0.053*	
C2	0.05773 (17)	0.67486 (15)	0.07315 (6)	0.0445 (3)	
H2A	−0.0442	0.6465	0.0429	0.053*	
H2B	0.1533	0.6131	0.0624	0.053*	
C6	0.47308 (16)	1.09143 (14)	0.12711 (7)	0.0442 (3)	
H6A	0.4868	1.0674	0.1755	0.053*	
H6B	0.5616	1.0388	0.1058	0.053*	
O7	0.05818 (13)	0.33529 (11)	0.09607 (6)	0.0573 (3)	
N3	−0.07066 (14)	0.28710 (12)	0.12031 (5)	0.0409 (3)	
N2	0.26751 (13)	0.30391 (12)	0.26544 (5)	0.0410 (3)	
O4	0.37816 (13)	0.37345 (12)	0.23659 (5)	0.0573 (3)	
O5	0.21779 (16)	0.35660 (13)	0.31806 (7)	0.0743 (4)	
O6	0.20595 (15)	0.18569 (12)	0.24225 (5)	0.0625 (3)	
N1	0.43842 (14)	0.14691 (14)	0.44408 (6)	0.0486 (3)	
O1	0.49718 (18)	0.26837 (13)	0.46985 (6)	0.0719 (3)	
O2	0.49348 (16)	0.10390 (15)	0.39153 (6)	0.0766 (4)	
O3	0.33557 (14)	0.07557 (17)	0.47326 (8)	0.0917 (5)	

### Atomic displacement parameters (Å^2^)

**Table:**

	*U*^11^	*U*^22^	*U*^33^	*U*^12^	*U*^13^	*U*^23^
O8	0.0685 (7)	0.0463 (6)	0.0767 (7)	−0.0175 (5)	0.0153 (6)	−0.0120 (5)
O9	0.0719 (7)	0.0479 (6)	0.0544 (6)	0.0080 (5)	0.0245 (5)	−0.0032 (4)
N5	0.0334 (5)	0.0314 (5)	0.0327 (5)	0.0000 (4)	0.0066 (4)	0.0000 (4)
N4	0.0540 (6)	0.0339 (5)	0.0495 (6)	−0.0102 (5)	0.0187 (5)	−0.0043 (4)
N6	0.0467 (6)	0.0392 (6)	0.0450 (6)	−0.0089 (4)	0.0112 (5)	−0.0005 (4)
C4	0.0464 (7)	0.0388 (6)	0.0310 (6)	−0.0053 (5)	0.0050 (5)	−0.0014 (4)
C1	0.0463 (7)	0.0442 (7)	0.0363 (6)	−0.0068 (5)	−0.0017 (5)	0.0017 (5)
C5	0.0439 (6)	0.0331 (6)	0.0420 (7)	−0.0028 (5)	0.0035 (5)	0.0057 (5)
C3	0.0580 (8)	0.0391 (7)	0.0377 (7)	0.0003 (6)	0.0109 (6)	0.0048 (5)
C2	0.0487 (7)	0.0451 (7)	0.0401 (7)	−0.0117 (6)	0.0066 (6)	−0.0092 (5)
C6	0.0389 (6)	0.0374 (7)	0.0565 (8)	−0.0016 (5)	0.0059 (6)	0.0043 (6)
O7	0.0538 (6)	0.0439 (5)	0.0785 (7)	−0.0018 (4)	0.0255 (5)	0.0070 (5)
N3	0.0442 (6)	0.0383 (6)	0.0402 (6)	0.0027 (5)	0.0050 (4)	0.0053 (4)
N2	0.0399 (5)	0.0419 (6)	0.0414 (6)	−0.0011 (4)	0.0059 (5)	−0.0061 (4)
O4	0.0572 (6)	0.0620 (7)	0.0559 (6)	−0.0157 (5)	0.0205 (5)	−0.0081 (5)
O5	0.0777 (7)	0.0731 (8)	0.0802 (8)	−0.0278 (6)	0.0434 (6)	−0.0406 (6)
O6	0.0849 (8)	0.0474 (6)	0.0571 (6)	−0.0186 (5)	0.0162 (6)	−0.0165 (5)
N1	0.0379 (5)	0.0574 (7)	0.0515 (7)	0.0028 (5)	0.0087 (5)	0.0133 (5)
O1	0.1058 (9)	0.0543 (7)	0.0572 (7)	−0.0008 (6)	0.0166 (6)	−0.0007 (5)
O2	0.0825 (8)	0.0947 (10)	0.0554 (7)	−0.0013 (7)	0.0206 (6)	−0.0126 (6)
O3	0.0447 (6)	0.1131 (11)	0.1208 (11)	−0.0054 (6)	0.0251 (7)	0.0587 (9)

### Geometric parameters (Å, °)

**Table:**

O8—N3	1.2411 (14)	C1—H1A	0.9700
O9—N3	1.2468 (14)	C1—H1B	0.9700
N5—C5	1.4918 (15)	C5—C6	1.5114 (18)
N5—C4	1.5000 (15)	C5—H5A	0.9700
N5—C1	1.5037 (15)	C5—H5B	0.9700
N5—H5	0.9100	C3—H3A	0.9700
N4—C2	1.4831 (16)	C3—H3B	0.9700
N4—C3	1.4878 (17)	C2—H2A	0.9700
N4—H4A	0.9000	C2—H2B	0.9700
N4—H4B	0.9000	C6—H6A	0.9700
N6—C6	1.4836 (16)	C6—H6B	0.9700
N6—H7A	0.8900	O7—N3	1.2379 (14)
N6—H7B	0.8900	N2—O6	1.2261 (14)
N6—H7C	0.8900	N2—O5	1.2402 (14)
C4—C3	1.5094 (17)	N2—O4	1.2512 (14)
C4—H4C	0.9700	N1—O3	1.2187 (15)
C4—H4D	0.9700	N1—O2	1.2269 (16)
C1—C2	1.5028 (19)	N1—O1	1.2611 (16)
			
C5—N5—C4	113.34 (9)	C6—C5—H5A	109.0
C5—N5—C1	109.08 (9)	N5—C5—H5B	109.0
C4—N5—C1	109.00 (9)	C6—C5—H5B	109.0
C5—N5—H5	108.4	H5A—C5—H5B	107.8
C4—N5—H5	108.4	N4—C3—C4	110.13 (10)
C1—N5—H5	108.4	N4—C3—H3A	109.6
C2—N4—C3	111.09 (9)	C4—C3—H3A	109.6
C2—N4—H4A	109.4	N4—C3—H3B	109.6
C3—N4—H4A	109.4	C4—C3—H3B	109.6
C2—N4—H4B	109.4	H3A—C3—H3B	108.1
C3—N4—H4B	109.4	N4—C2—C1	109.54 (10)
H4A—N4—H4B	108.0	N4—C2—H2A	109.8
C6—N6—H7A	109.5	C1—C2—H2A	109.8
C6—N6—H7B	109.5	N4—C2—H2B	109.8
H7A—N6—H7B	109.5	C1—C2—H2B	109.8
C6—N6—H7C	109.5	H2A—C2—H2B	108.2
H7A—N6—H7C	109.5	N6—C6—C5	108.72 (10)
H7B—N6—H7C	109.5	N6—C6—H6A	109.9
N5—C4—C3	111.21 (10)	C5—C6—H6A	109.9
N5—C4—H4C	109.4	N6—C6—H6B	109.9
C3—C4—H4C	109.4	C5—C6—H6B	109.9
N5—C4—H4D	109.4	H6A—C6—H6B	108.3
C3—C4—H4D	109.4	O7—N3—O8	120.31 (11)
H4C—C4—H4D	108.0	O7—N3—O9	120.08 (11)
C2—C1—N5	110.85 (10)	O8—N3—O9	119.59 (11)
C2—C1—H1A	109.5	O6—N2—O5	119.43 (11)
N5—C1—H1A	109.5	O6—N2—O4	121.27 (11)
C2—C1—H1B	109.5	O5—N2—O4	119.29 (11)
N5—C1—H1B	109.5	O3—N1—O2	123.17 (15)
H1A—C1—H1B	108.1	O3—N1—O1	119.22 (14)
N5—C5—C6	113.14 (10)	O2—N1—O1	117.55 (12)
N5—C5—H5A	109.0		
			
C5—N5—C4—C3	178.55 (10)	C2—N4—C3—C4	57.49 (14)
C1—N5—C4—C3	56.88 (13)	N5—C4—C3—N4	−56.80 (14)
C5—N5—C1—C2	177.55 (10)	C3—N4—C2—C1	−58.69 (14)
C4—N5—C1—C2	−58.23 (13)	N5—C1—C2—N4	59.29 (14)
C4—N5—C5—C6	71.34 (13)	N5—C5—C6—N6	−172.78 (10)
C1—N5—C5—C6	−167.04 (11)		

### Hydrogen-bond geometry (Å, °)

**Table:**

*D*—H···*A*	*D*—H	H···*A*	*D*···*A*	*D*—H···*A*
N5—H5···O1^i^	0.91	2.00	2.8074 (16)	146.
N5—H5···O2^i^	0.91	2.34	3.1755 (17)	152.
N4—H4A···O9	0.90	2.05	2.9008 (15)	158.
N4—H4A···O7	0.90	2.29	2.9947 (15)	135.
N4—H4B···O5^ii^	0.90	1.93	2.8065 (16)	165.
N4—H4B···O6^ii^	0.90	2.43	3.0387 (15)	125.
N6—H7A···O1^iii^	0.89	2.16	2.9224 (15)	144.
N6—H7A···O3^iii^	0.89	2.47	3.322 (2)	161.
N6—H7B···O9^iv^	0.89	2.15	3.0360 (15)	173.
N6—H7B···O8^iv^	0.89	2.45	3.1171 (16)	132.
N6—H7C···O4^v^	0.89	1.99	2.8033 (14)	152.

Symmetry codes: (i) −*x*+1, *y*+1/2, −*z*+1/2; (ii) −*x*, *y*+1/2, −*z*+1/2; (iii) *x*, −*y*+3/2, *z*−1/2; (iv) *x*+1, *y*+1, *z*; (v) *x*, *y*+1, *z*.
